# Supplementary material for: CAR T-cells targeting FGFR4 and CD276 simultaneously show potent antitumor effect against childhood rhabdomyosarcoma
Source: Nat Commun. 2024 Jul 23;15:6222. doi: 10.1038/s41467-024-50251-x (PMC11266617; doi:10.1038/s41467-024-50251-x)
Supplement: Supplementary file 1 — Supplementary Information [file 41467_2024_50251_MOESM1_ESM.pdf]

Supplementary Information

CAR T-cells targeting FGFR4 and CD276 simultaneously show potent antitumor effect against childhood rhabdomyosarcoma

Meijie Tian, Jun S. Wei, Adam Tai-Chi Cheuk, David Milewski, Zhongmei Zhang, Yong Yean Kim, Hsien-Chao Chou, Can Liu, Sherif Badr, Eleanor G. Pope, Abdelrahman Rahmy, Jerry T.Wu, Michael C. Kelly, Xinyu Wen, and Javed Khan

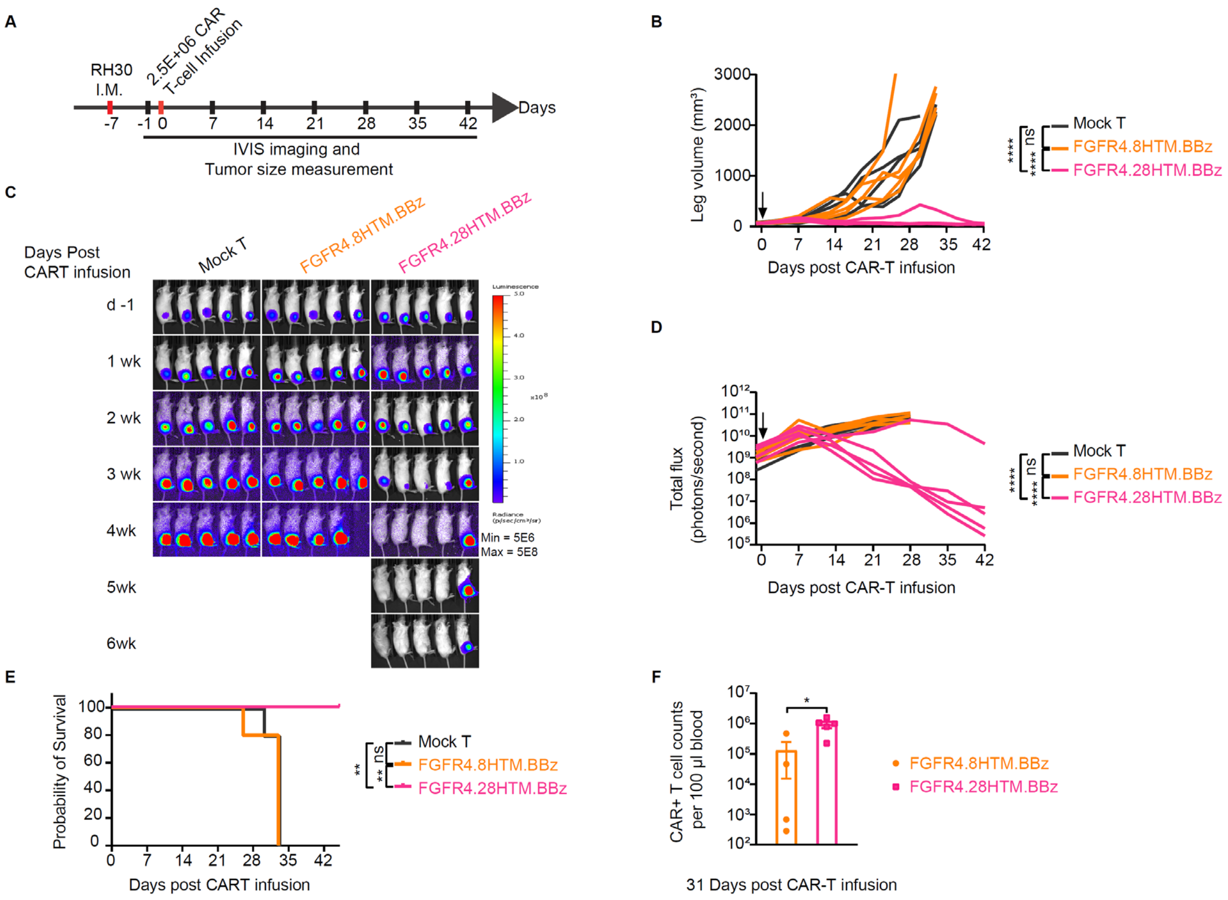

Supplementary Fig. 1 (related to Fig. 1). Low dose (2.5E+6) of FGFR4-CAR T-cells containing a CD28 HTM outperformed the original design with a CD8 HTM in RH30 orthotopic xenografts.

(**A**) Schema of RH30 orthotopic, intramuscular, xenograft model infused with mock, or 2.5E6 FGFR4.8HTM.BBz, or FGFR4.28HTM.BBz CAR T-cells on day 7 post tumor inoculation. (**B**) tumor size, (**C**) Bioluminescent images, and (**D**) total flux of RH30 intramuscular xenografts growth before (day -1) and after infusion with mock T-cells, FGFR4.8HTM.BBz, or FGFR4.28HTM.BBz CAR T-cells. Each replicate per group is shown, $n = 5$. Mixed-effects analysis or Two-way repeated measures (RM) ANOVA analysis is used to calculate the p values between two groups in Figures B and D. ****$p < 0.0001$, ns, not significant. (**E**) Kaplan-Meier survival analysis of mice receiving different treatments is shown. **$p = 0.0035$; ns, not significant; by log-rank test. (**F**) FGFR4 CAR T-cell counts per 100μl in blood from mice of RH30 orthotopic model at day 31 post-treated with 2.5E+6 CAR T-cells. Statistics for comparing two FGFR4 CAR T-cell counts represent two-tailed unpaired Mann-Whitney test. *$p = 0.0317$. Source data are provided as a Source Data file.

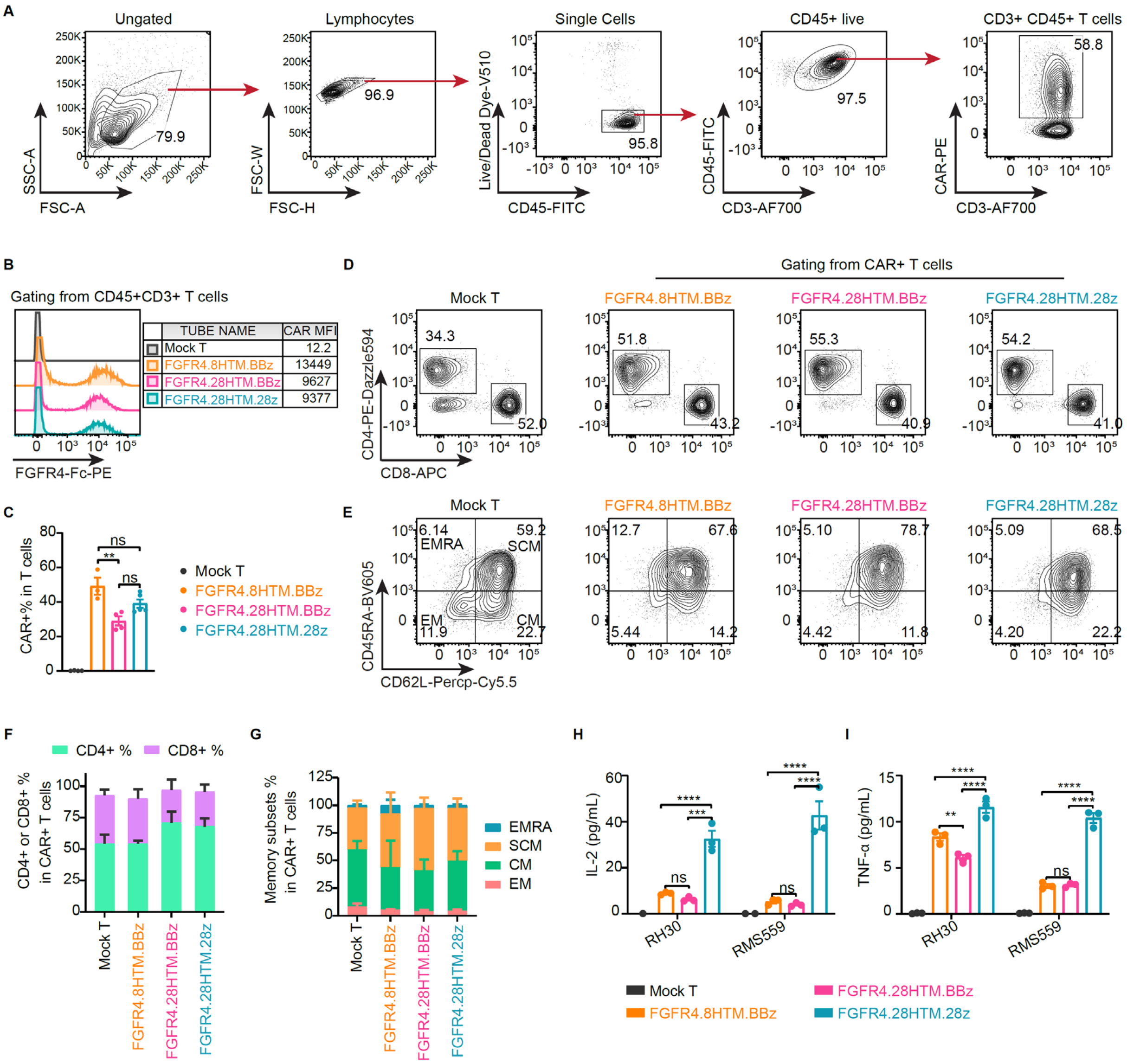

Supplementary Fig. 2 (related to Fig. 1). Phenotypic characterization of three FGFR4 CAR T-cells before infusion into tumor-bearing mice. (**A**) Flow cytometry-based gating strategy for analyzing the phenotypes of CAR+ T cells presented on Fig. 1L, S1F, 3K, 4F, 4J, 4K, S2, S5, S6, and S7G. (**B**) Representative histogram of 3 FGFR4 CAR expressions on T-cell surface assessed by binding to FGFR4-Fc protein after 9 days post-transduction. Mean fluorescent intensity (MFI) of CAR expression in all three FGFR4-CAR constructs and mock T-cells (in black) shown in the table. (**C**) Percentage of CAR positive in all $CD45^{+}CD3^{+}$ T-cells measured by flow cytometry at day 9 after manufacturing. Data represent independent experiments with 4 different T-cell donors, mean ± SEM. Statistics represent one-way ANOVA with Tukey's multiple comparisons. $^{**}p = 0.0058$; ns, not significant. (**D** and **E**) Representative flow cytometry plots characterize phenotypes of CAR-transduced T-cells, including $CD4^{+}$ and $CD8^{+}$ T-

cells, stem cell memory (SCM, $CD45RA^+$ and $CD62L^+$), central memory (CM, $CD45RA^-$ and $CD62L^+$), effector memory (EM, $CD45RA^-$ and $CD62L^-$) and terminally differentiated effector memory (EMRA, $CD45RA^+$ and $CD62L^-$) on day 10 after CAR transduction. (**F** and **G**) Mean frequencies were plotted for $CD4^+$ and $CD8^+$ T-cells for panel D, or memory subsets for panel E with SEM for 4 independent experiments. (**H** and **I**) IL-2 (H) and TNF- α (I) released by three FGFR4 CAR T-cells following a 72-hour coculture with RH30 or RMS559 cells. Data means are plotted with SEM for 3 independent cocultures. ***p = 0.0002, ****p < 0.0001 in H; **p = 0.0018, ****p < 0.0001 in I, and ns, not significant, by two-way ANOVA with Tukey's multiple-comparison test. Source data are provided as a Source Data file.

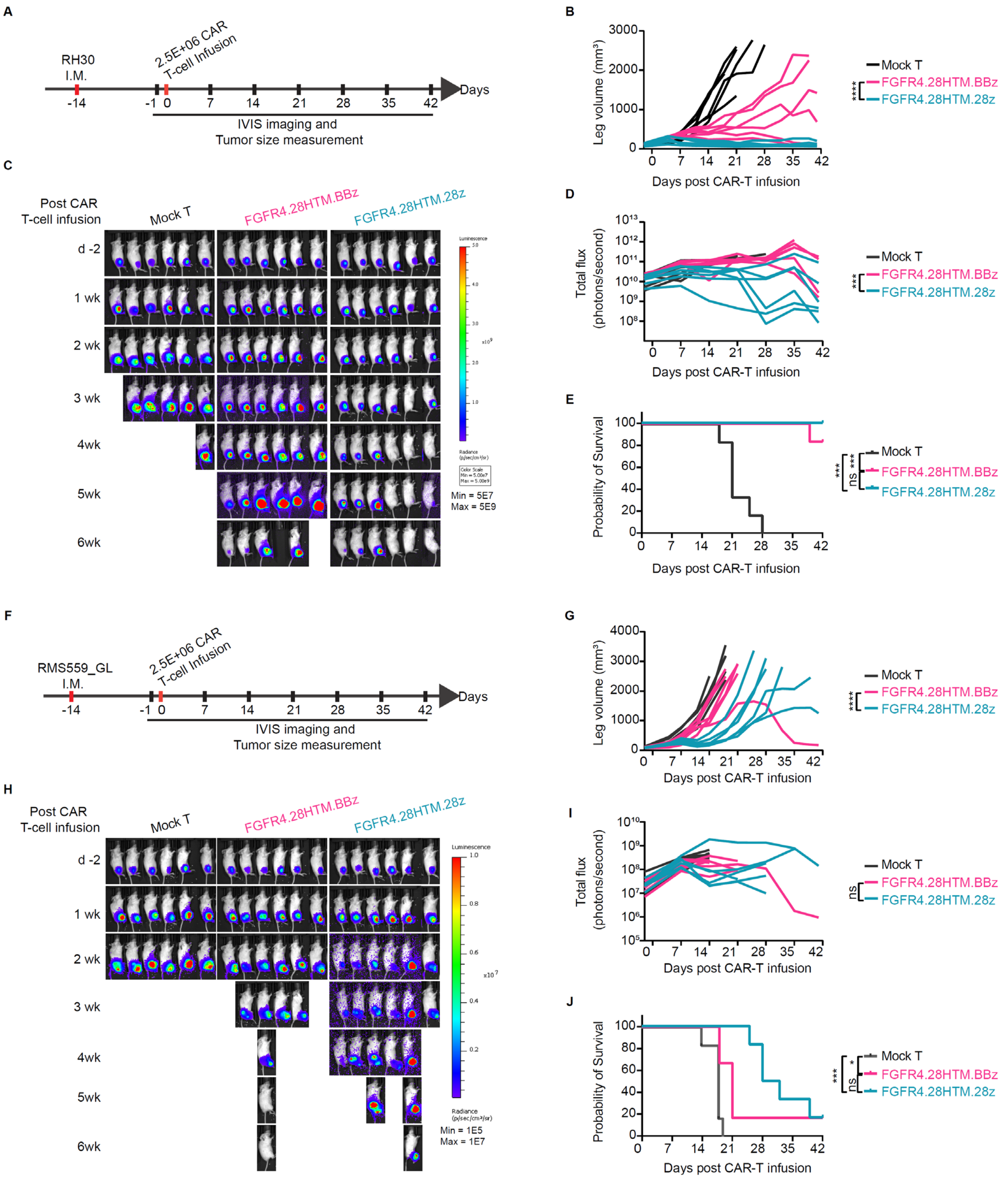

Supplementary Fig. 3 (related to Fig. 1). Altering the HTM and CSD significantly improves the killing ability of FGFR4 CAR T-cells from donor 5 against two RMS orthotopic xenografts. (**A**) Schema of testing CAR T-cells in an RH30 intramuscular (I.M.) xenograft model infused with mock or 2.5E6 FGFR4 CAR T-cells derived from donor 5, 14 days after tumor inoculation. (**B**) Leg volume for testing CAR T-cell efficacy in an RH30 I.M. xenograft model. Each line represents an individual mouse (*n* = 6 per group).

Mixed effects analysis is used to calculate the P values between two groups. ****$p < 0.0001$. (**C** and **D**) RH30 tumor burden, represented by bioluminescent images (**C**) or total flux (photons/second, **D**), was assessed by an IVIS imaging system. Mixed effects analysis was used to calculate the *p*-value between two groups. ***$p = 0.0002$. (**E**) Kaplan-Meier survival analysis of RH30-bearing mice treated with mock or FGFR4 CAR T-cells ($n = 6$ mice/group). ***$p = 0.0005$, ns for not significant, by log-rank test. (**F**) Schema of testing the activity of 2.5E6 FGFR4 CAR T-cells from donor 5 in an RMS559 I.M. xenograft mouse model. (**G**) Tumor size was measured as leg volume. Each line represents an individual mouse ($n = 6$ per group). ****$p < 0.0001$; ns, not significant, by mixed-effects analysis between two groups. (**H** and **I**) RMS559 tumor burden, represented by bioluminescent images (**H**) and total flux (photons/second, **I**), was assessed by an IVIS imaging system. Mixed-effects analysis was used to calculate p-values between the two groups, respectively. (**J**) Kaplan-Meier survival analysis of mice treated with mock T or FGFR4 CAR T-cells ($n = 6$ mice/group) by log-rank test. *$p = 0.0166$, ***$p = 0.0013$; ns, not significant. Source data are provided as a Source Data file.

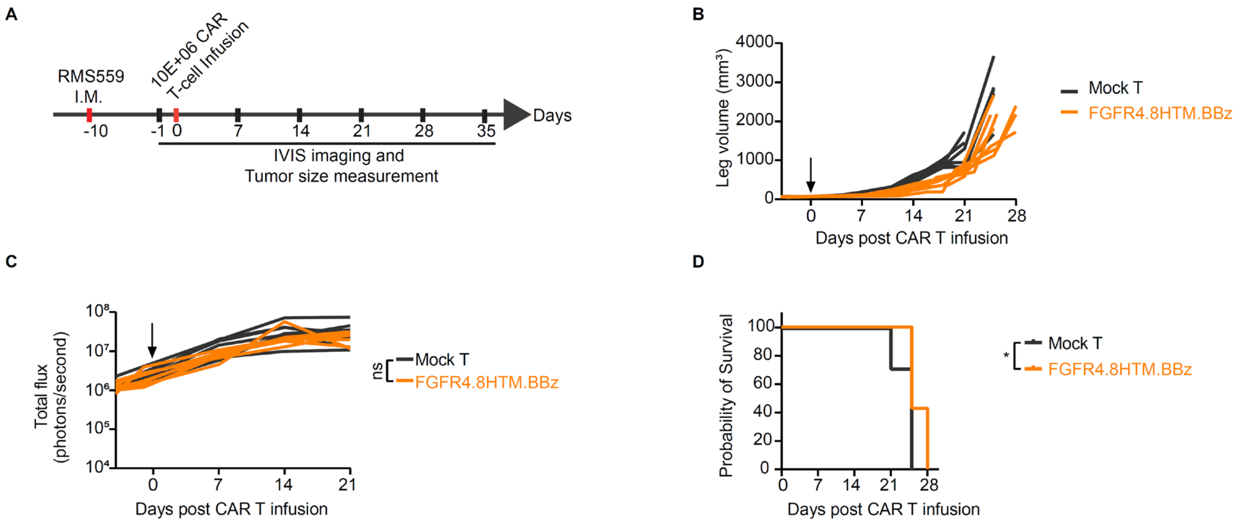

Supplementary Fig. 4 (related to Fig. 1). 10E+6 FGFR4-CAR T-cells incorporating a CD8 HTM and 4-1BB CSD showed minimal activity in RMS559 orthotopic xenografts. (**A**) Schema of the RMS559 intramuscular xenograft model infused with mock or 10E+6 FGFR4.8HTM.BBz CAR T-cells on day 10 post-tumor inoculation. (**B**) Tumor size and (**C**) total flux of RMS559 intramuscular xenografts growth before (day -1, day -4) and after infusion with mock T-cells or FGFR4.8HTM.BBz CAR T-cells. Each replicate per group is shown, $n = 7$. Two-way repeated measures (RM) ANOVA analysis is used to calculate the p values between two groups: ns, not significant. (**D**) Kaplan-Meier survival analysis of mice receiving different treatments is shown. $^{*}p = 0.0287$, by log-rank test. Source data are provided as a Source Data file.

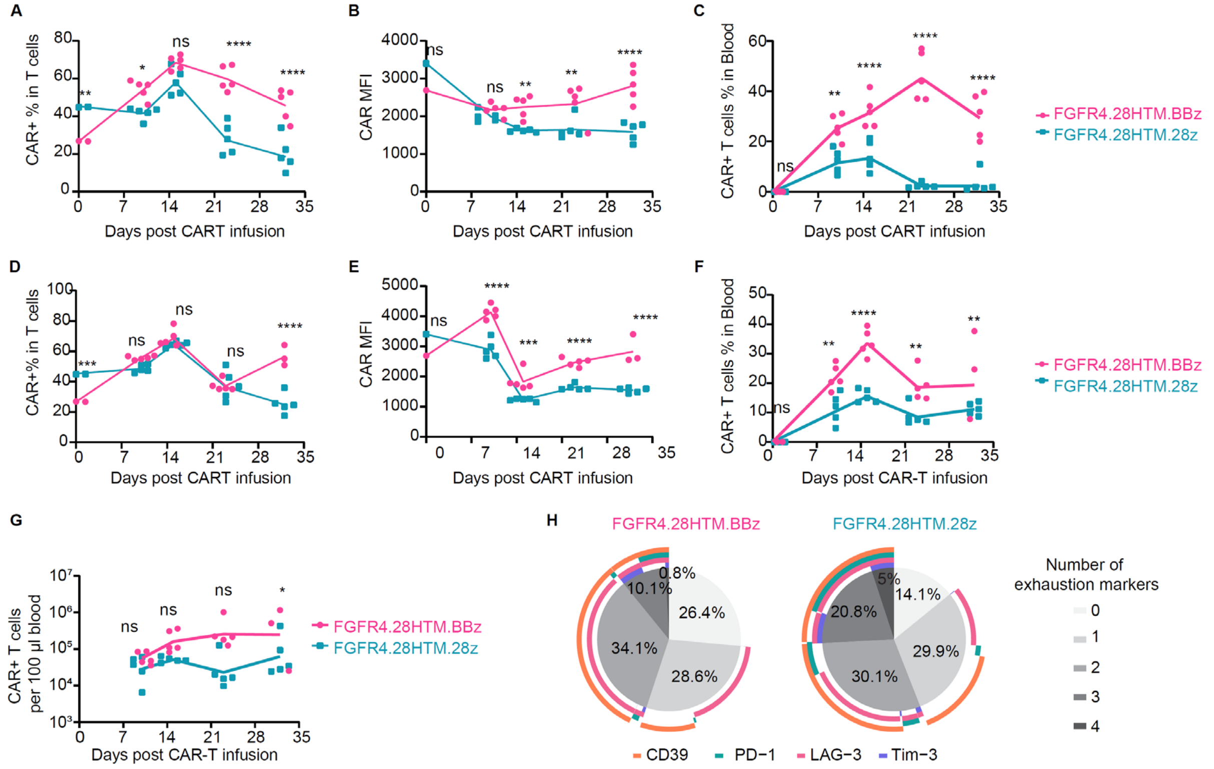

Supplementary Fig. 5 (related to Fig. 1). Phenotypic characterization of FGFR4 CAR T-cells in vivo. (**A-C**) Percentage of CAR$^+$ in CD45$^+$CD3$^+$ T-cells from blood (**A**), MFI of surface CAR expression (**B**), and the percentage of CAR$^+$ T-cells in blood singlets (**C**) from mice of RH30 I.M. model treated with 2.5E6 CAR T-cells analyzed by flow cytometry at day 10, day 15, day 23, and day 32 ($n = 5$; each replicate is shown). *$p = 0.0166$, ** $p = 0.0049$ in A; ** $p = 0.0096$ for day 15, ** $p = 0.0037$ for day 23 in B; **$p = 0.0022$ in C; ****$p < 0.0001$, ns, not significant, by two-way ANOVA with Sidak's multiple comparisons test. (**D-F**) Percentage of CAR$^+$ in CD45$^+$CD3$^+$ T-cells (**D**), MFI of T-cell surface CAR expression (**E**), and the percentage of CAR$^+$ T-cells in the blood (**F**) at day 10, 15, 23, or 32 after CAR T-cell infusion into RMS559 I.M xenografts bearing mice ($n = 5$ mice per group). Two-way ANOVA with Sidak's multiple comparisons test is used to calculate the above p values. ***$p = 0.0004$ in D; ***$p = 0.0009$ in E; **$p = 0.0068$ for day 10, **$p = 0.01$ for day 23, **$p = 0.0074$ for day 32 in F; ****$p < 0.0001$, ns, not significant. (**G**) Expansion dynamics of FGFR4 CAR T-cell counts per 100µl in blood from mice of RMS559 I.M. model treated with 2.5E6 CAR T-cells by flow cytometry. Statistics for comparing two FGFR4 CAR T-cell counts at each time point represent two-way ANOVA with Sidak's multiple-comparison test. *$p = 0.0348$; ns, not significant. (**H**) Percentage of CAR T-cells expressing or co-expressing CD39, PD-1, LAG-3, and TIM-3 at day 32 after T-cell infusion into RMS559-bearing mice. Source data are provided as a Source Data file.

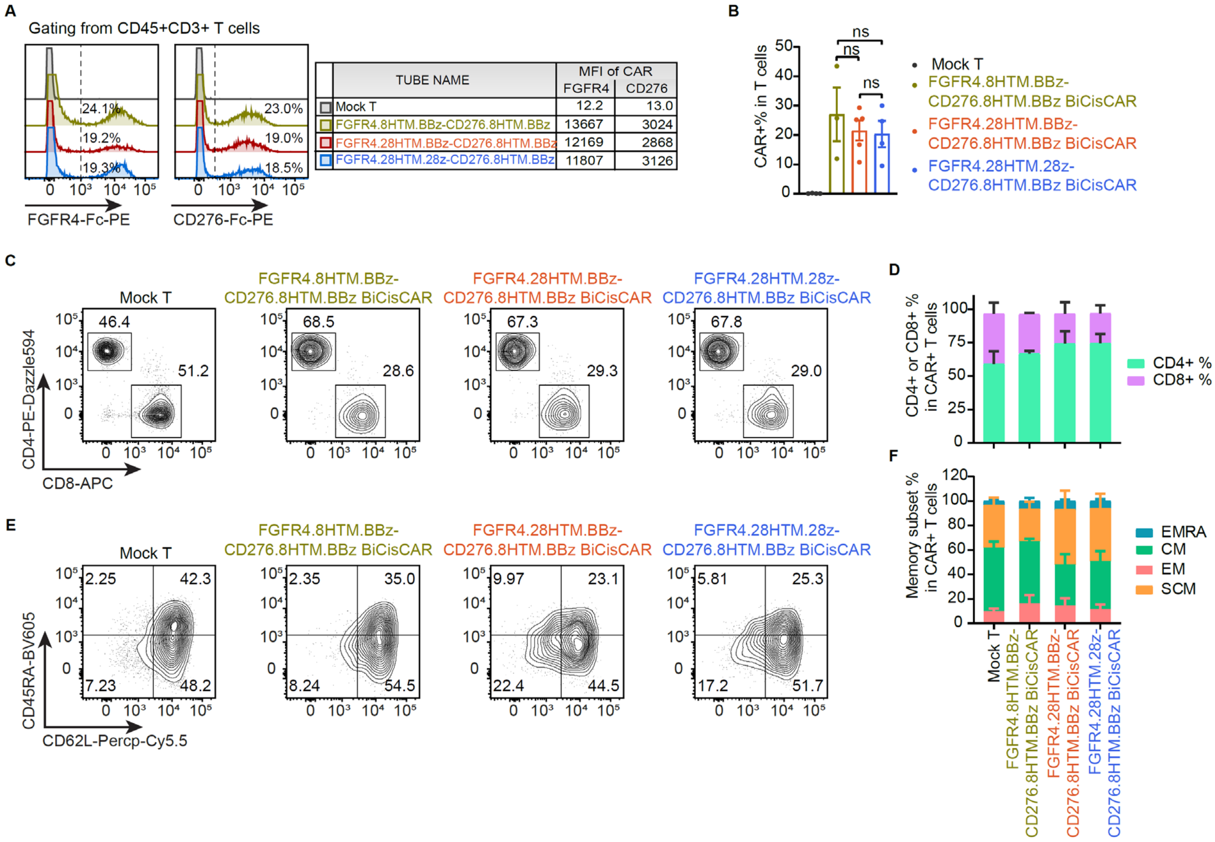

Supplementary Fig. 6 (related to Fig. 3). Phenotypic characterization of three BiCisCAR T-cells before infusion into orthotopic RH30-bearing mice. (**A**) Representative histogram of CAR cell surface expression among different BiCisCAR constructs assessed by binding to FGFR4-Fc or CD276-Fc protein after manufacturing for 10 days. Mean fluorescent intensity (MFI) of CAR expression in all three BiCisCAR constructs, and mock T-cells (shown in black) are shown in the right table. (**B**) Mean percentage ± SEM of CAR positive in all $CD45^{+}CD3^{+}$ T-cells measured by flow cytometry at day 10 after manufacturing. Data represent independent experiments with 3 or 5 different T-cell donors. Statistics represents one-way ANOVA with Tukey's multiple comparison tests. Ns, not significant. (**C** and **E**) Representative flow cytometry plots characterize phenotypes of CAR-transduced T-cells, including $CD4^{+}$ and $CD8^{+}$ T-cells, stem cell memory (SCM, $CD45RA^{+}$ and $CD62L^{+}$), central memory (CM, $CD45RA^{-}$ and $CD62L^{+}$), effector memory (EM, $CD45RA^{-}$ and $CD62L^{-}$) and terminally differentiated effector memory (EMRA, $CD45RA^{+}$ and $CD62L^{-}$) on day 10 after CAR transduction. (**D** and **F**) Mean frequencies were plotted for $CD4^{+}$ and $CD8^{+}$ T-cells for panel C, or memory subsets for panel E with SEM for 3 or 5 independent experiments. Source data are provided as a Source Data file.

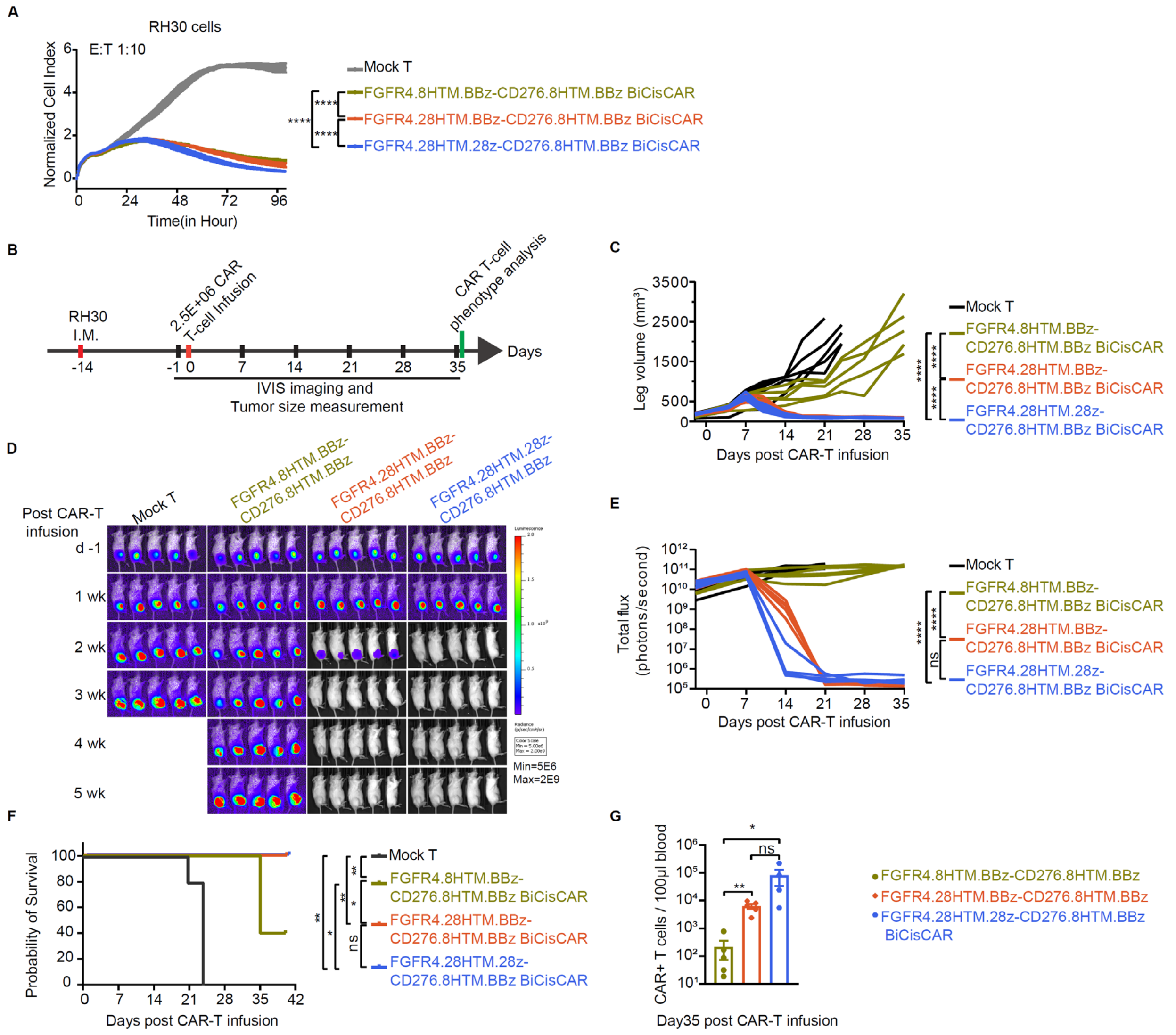

Supplementary Fig. 7 (related to Fig. 3). Dual targeting CAR T-cells using the same CD8 HTM and 4-1BB CSD construct for FGFR4 and CD276 exhibit reduced tumor killing against RH30 orthotopic model.(**A**) Cytolytic activity of three BiCisCAR T-cells against RH30 at an E:T ratio of 1:10 by an xCELLigence RTCA. Statistical analysis was performed with two-way RM ANOVA. ****$p < 0.0001$. (**B**) Schema of an *in vivo* model testing the activity of 2.5E6 BiCisCAR T-cells against an RH30 I.M. xenograft. (**C**) Tumor volumes following CAR T-cell infusion. Data indicates the tumor volume of each mouse (n = 5). ****$p < 0.0001$, as determined by two-way RM ANOVA. (**D** and **E**) Bioluminescence images (**D**) and total flux (**E**) of RH30 tumor growth assessed by IVIS imaging post three BiCisCAR T-cells treatment. A two-way RM analysis was used to calculate the p-value between every two BiCisCAR T-cells groups respectively. ****$p < 0.0001$. (**F**) In the above model, the Kaplan-Meier survival curve of mice (n = 5 mice per group); comparisons of survival curves was determined by log-rank test, *p = 0.0495, **p = 0.0035. (**G**) Cell counts of three BiCisCAR T-cells per 100µl blood from mice treated with 2.5E6 CAR T-cells were

analyzed by flow cytometry at day 35 ($n = 5$). Statistics for every two BiCisCAR T-cell counts represent a two-tail nonparametric test using the Mann-Whitney test. *$p = 0.0159$, **$p = 0.0079$, ns, not significant. Source data are provided as a Source Data file.

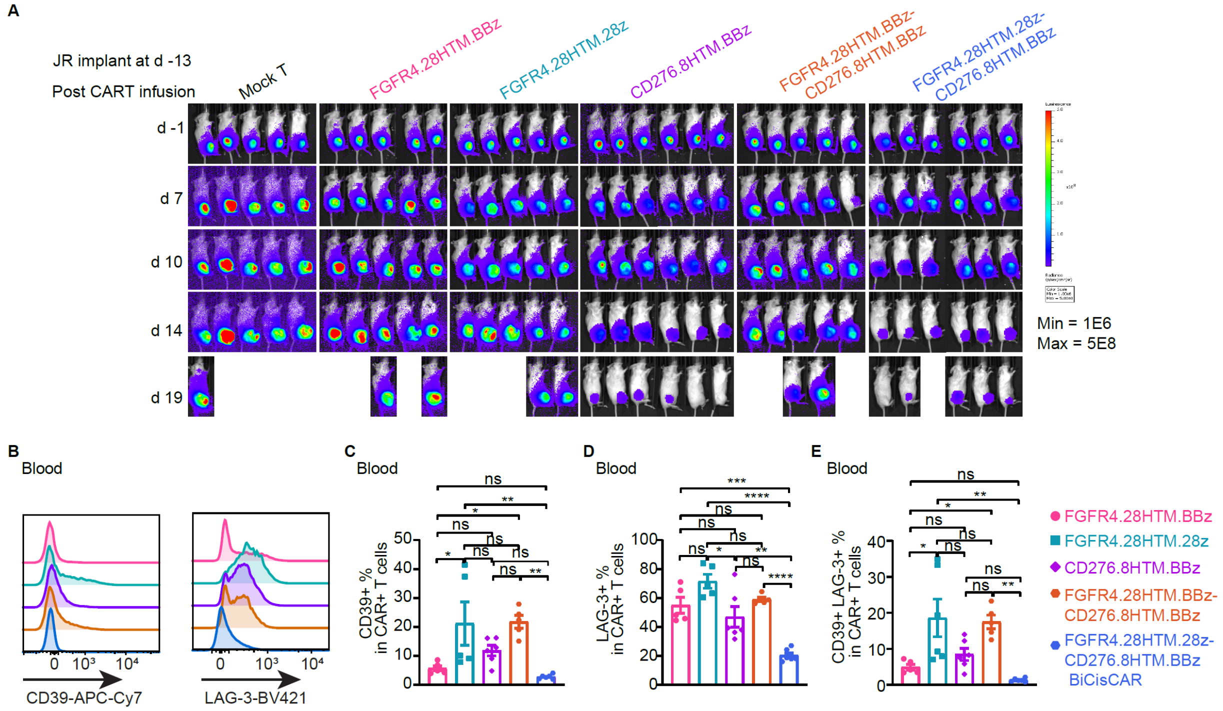

Supplementary Fig. 8 (related to Fig. 4) Dual targeting BiCisCAR with two different CSDs exhibited rapid tumor shrinkage and limited exhaustion. (**A**) Representative tumor BLI images of JR_Luc tumor growth in the orthotopic xenograft model shown in Fig. 4. (**B**) Representative flow cytometry illustrating CD39 and LAG-3 expression in CAR$^+$ T-cells in the blood of mice from the JR I.M xenograft model, 21 days after infusion of 2.5E+6 CAR T-cells. (**C** - **E**) Percentages of CD39$^+$ (**C**), LAG-3$^+$ (**D**), and CD39$^+$ LAG-3$^+$ (**E**) in CAR$^+$ T-cells circulating in the blood from mice 21 days after CAR T-cells infusion ($n = 5$ or 6, mean ± SEM). *$p < 0.05$, **$p < 0.01$, ***$p < 0.001$, and ****$p < 0.0001$, by one-way ANOVA, with Tukey's multiple comparison tests, the full list of p values can be found in the Source Data. Source data are provided as a Source Data file.

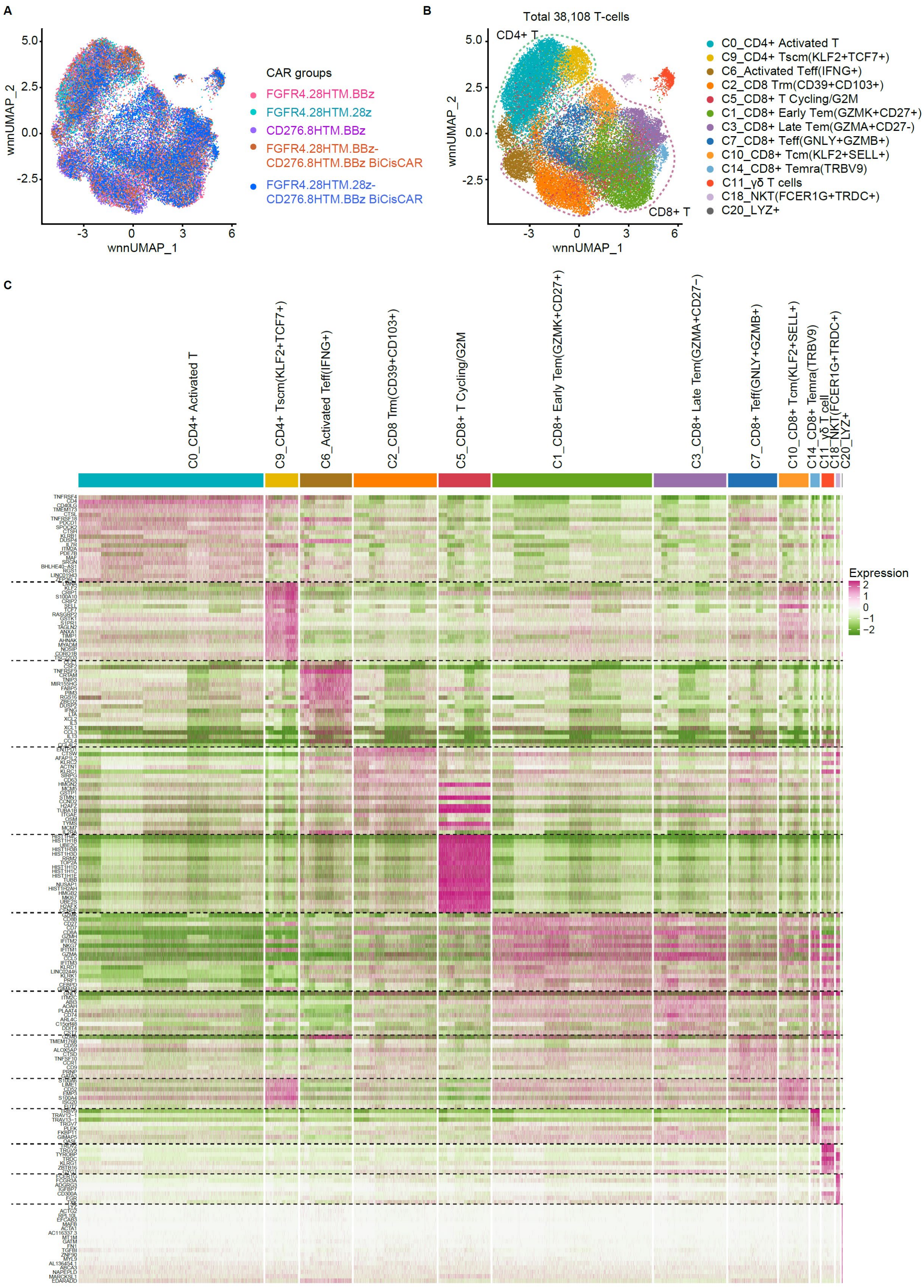
A
B
C
Total 38,108 T-cells
CD4+ T
CD8+ T
wnnUMAP_1
wnnUMAP_2
CAR groups
FGFR4.28HTM.BBz
FGFR4.28HTM.28z
CD276.8HTM.BBz
FGFR4.28HTM.BBz-CD276.8HTM.BBz BiCisCAR
FGFR4.28HTM.28z-CD276.8HTM.BBz BiCisCAR
C0_CD4+ Activated T
C9_CD4+ Tscm(KLF2+TCF7+)
C6_Activated Teff(IFNG+)
C2_CD8 Trm(CD39+CD103+)
C5_CD8+ T Cycling/G2M
C1_CD8+ Early Tem(GZMK+CD27+)
C3_CD8+ Late Tem(GZMA+CD27-)
C7_CD8+ Teff(GNLY+GZMB+)
C10_CD8+ Tcm(KLF2+SELL+)
C14_CD8+ Temra(TRBV9)
C11_γδ T cells
C18_NKT(FCER1G+TRDC+)
C20_LYZ+
Expression

Supplementary Fig. 9 (related to Fig. 5). Cluster annotation based on the top genes of each cluster. (**A**) UMAP visualization of single cells from five CAR T-cell groups based on WNN assay after removing batch effect among sequencing lanes by SCT. Cells are colored according to CAR T-cell groups. (**B**) WNN UMAP visualization of a total of 38,108 tumor-infiltrating T-cells from the above model, revealed 13 clusters with different transcriptome and protein profiles. The green dash circle gates out the $CD4^+$ T-cells and the purple dash circle frames out $CD8^+$ T-cells. (**C**) Single cell heatmap showing the top 20 differentially expressed genes of each cluster for cell type annotation.

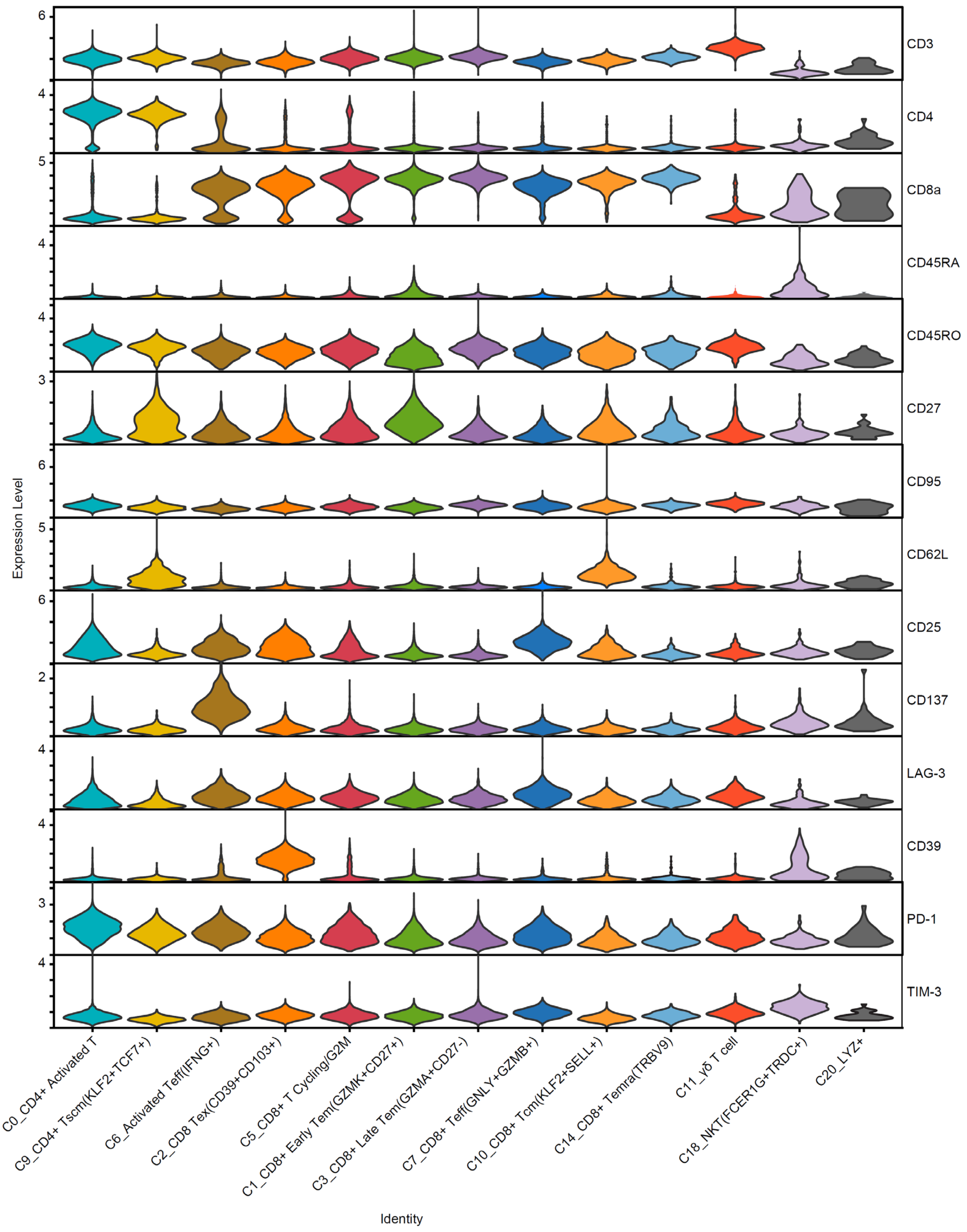

Supplementary Fig. 10 (related to Fig. 5). Violin plots of surface protein markers expression levels among 13 clusters were used for cluster annotation.

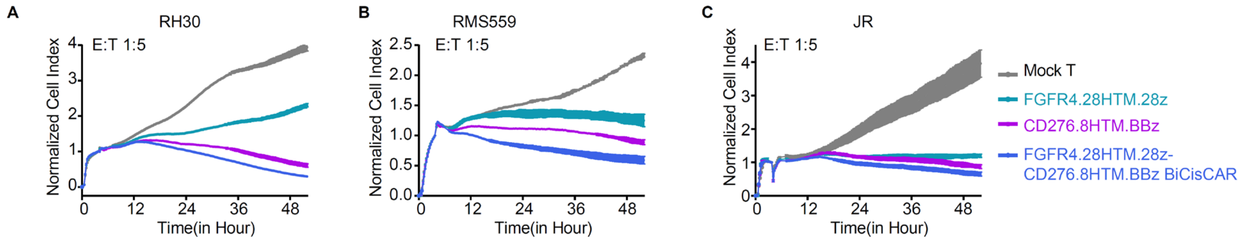

Supplementary Fig. 11 (related to Fig. 7). FGFR4.28HTM.28z-CD276.8HTM.BBz BiCisCAR T-cells exhibited an additive or synergistic effect on cytolytic activity. (A to **C)** Cytolytic activities of FGFR4.28HTM.28z, CD276.8HTM.BBz CARs or FGFR4.28HTM.28z-CD276.8HTM.BBz BiCisCAR T-cells were evaluated in vitro with RTCA against RH30 (A), RMS559 (B), or JR (C) cells. Source data are provided as a Source Data file.

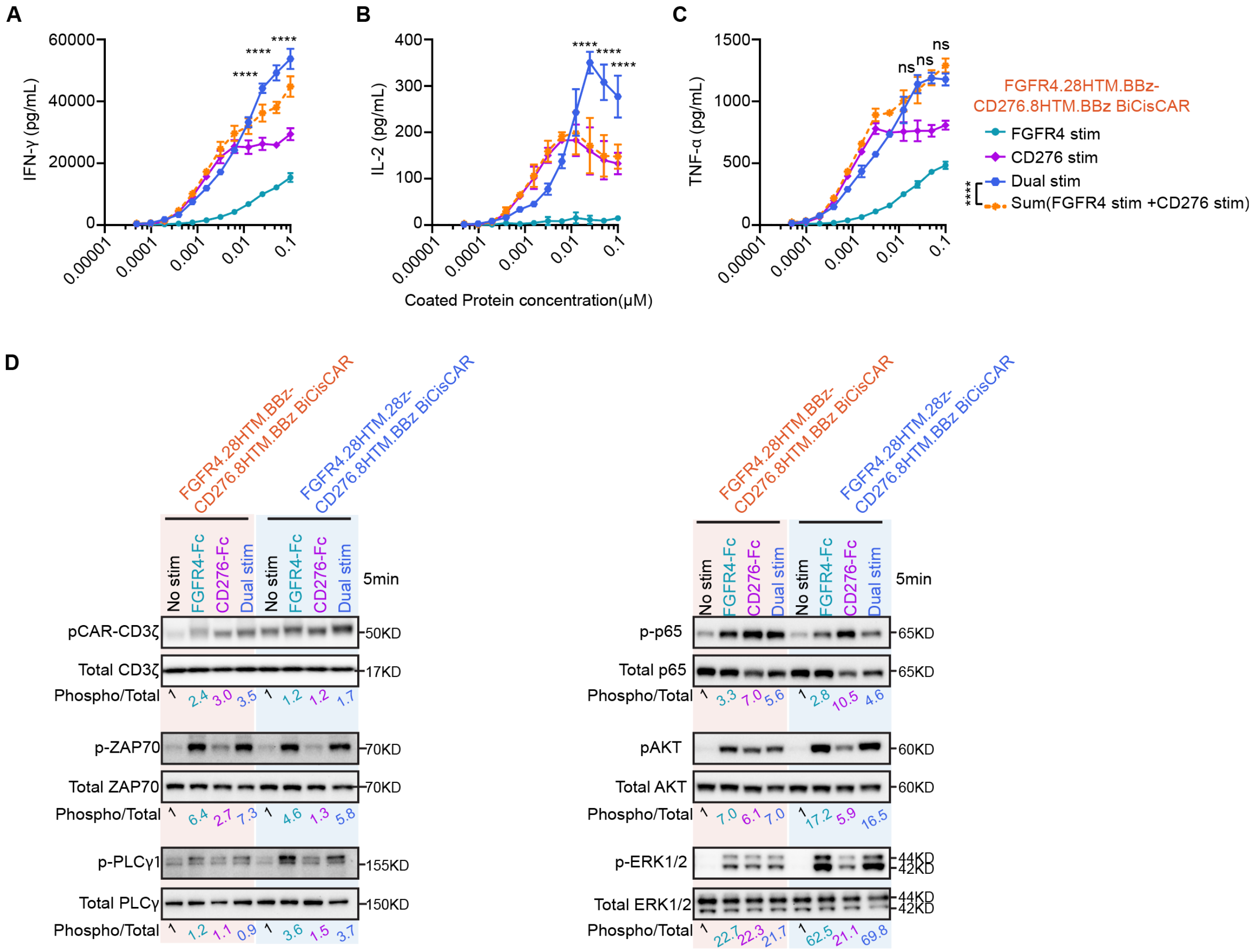

Supplementary Fig. 12 (related to Fig. 7) FGFR4 and CD276 dual-targeting CAR T-cells with 4-1BB CSDs demonstrate suboptimal downstream T-cell activation signaling. (**A**) IFN-γ, **(B)** IL-2 (B), and **(C)** TNF-α release by FGFR4.28HTM.BBz-CD276.8HTM.BBz BiCisCAR T-cells following a 20-hour stimulation with plate-coated FGFR4-Fc, CD276-Fc, or both proteins. Data are shown as the mean ± SD; $n = 3$ independent stimulation with CAR T-cells. The green dotted line shows the sum of cytokine released after single protein stimulation. Two-way ANOVA Sidak's multiple comparisons test was performed to statistic the difference between two proteins' dual stimulation and the sum of single stimulation. ****$p <$ 0.0001; ns, not significant. (**D**) Time course of CAR-CD3ζ, ZAP70, PLCγ1, p65, Akt and Erk1/2 phosphorylation in FGFR4.28HTM.BBz-CD276.8HTM.BBz BiCisCAR or FGFR4.28HTM.28z-CD276.8HTM.BBz BiCisCAR T-cells after CAR cross-linking (FGFR4-Fc protein for FGFR4 CAR, CD276-Fc protein for B7-H3, or both proteins for dual CAR cross-linking) measured by Western blot analysis. Numbers under the gels represent the ratio of the intensity of the signal obtained with phospho-specific antibodies relative to the total. Relative values were normalized to one of the unstimulated controls. Source data are provided as a Source Data file.

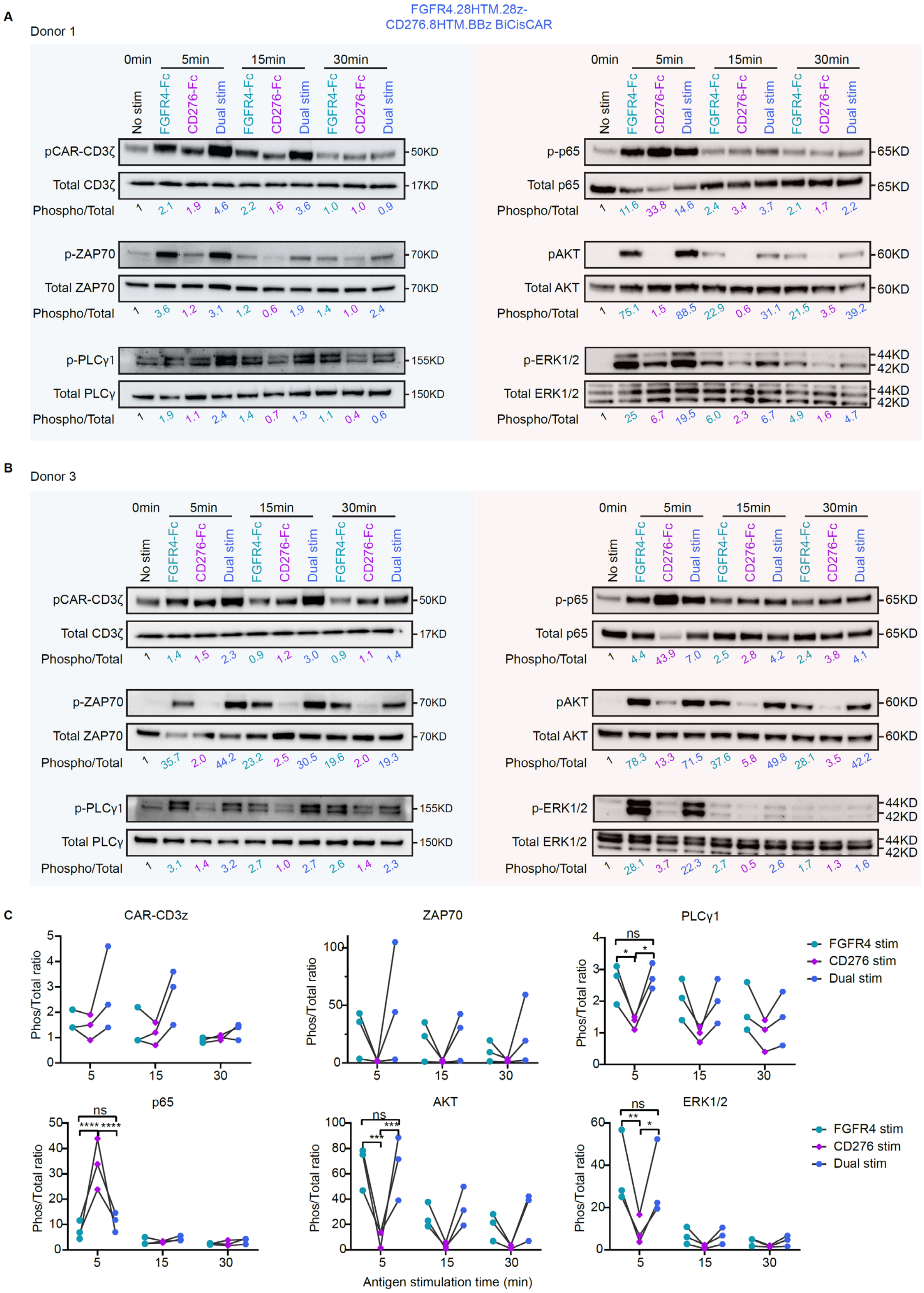
A
Donor 1
FGFR4.28HTM.28z-
CD276.8HTM.BBz BiCisCAR
0min
5min
15min
30min
No stim
FGFR4-Fc
CD276-Fc
Dual stim
pCAR-CD3ζ
Total CD3ζ
Phospho/Total
p-ZAP70
Total ZAP70
p-PLCγ1
Total PLCγ
p-p65
Total p65
pAKT
Total AKT
p-ERK1/2
Total ERK1/2
50KD
17KD
70KD
155KD
150KD
65KD
60KD
44KD
42KD
B
Donor 3
C
CAR-CD3z
ZAP70
PLCγ1
p65
AKT
ERK1/2
Phos/Total ratio
Antigen stimulation time (min)
FGFR4 stim
CD276 stim
Dual stim
ns

Supplementary Fig. 13 (related to Fig. 7). Western blot analyses of dual-targeting CAR T-cells using different CSDs demonstrate elevated downstream T-cell activation signaling by exposure to both antigens. (**A** and **B**) Time course of CAR-CD3ζ, ZAP70, PLCγ1, p65, Akt and Erk1/2 phosphorylation in FGFR4.28HTM.28z-CD276.8HTM.BBz BiCisCAR T-cells after stimulation by indicated antigens using T cells from another 2 independent donors. (**C**) Dot plots depict the ratio of immune band intensities between phospho-protein and total protein from three independent experiments using T-cells from 3 donors. Data from the same donor are connected by black lines. Two-way ANOVA Tukey's multiple comparison tests were performed between two paired conditions. *$p \leq 0.05$, **$p \leq 0.01$, ***$p \leq 0.001$, ****$p \leq 0.0001$, the full list of p values can be found in the Source Data. Source data are provided as a Source Data file.

Supplementary Table 1

Antibody-oligo conjugates directed against T-cell antigens and cell hashing antibodies were used in CITE-seq assay

CITE-seq Antibody name	Surface marker	isotype	Clone	Catalog #
TotalSeq™-C0034 anti-human CD3 Antibody	CD3	Mouse IgG1, κ	UCHT1	300479
TotalSeq™-C0072 anti-human CD4 Antibody	CD4	Mouse IgG1, κ	RPA-T4	300567
TotalSeq™-C0046 anti-human CD8 Antibody	CD8α	Mouse IgG1, κ	RPA-T8	344753
TotalSeq™-C0063 anti-human CD45RA Antibody	CD45RA	Mouse IgG2b, κ	HI100	304163
TotalSeq™-C0087 anti-human CD45RO Antibody	CD45RO	Mouse IgG2a, κ	UCHL1	304259
TotalSeq™-C0154 anti-human CD27 Antibody	CD27	Mouse IgG1, κ	O323	302853
TotalSeq™-C0156 anti-human CD95 (Fas) Antibody	CD95	Mouse IgG1, κ	DX2	305651
TotalSeq™-C0147 anti-human CD62L Antibody	CD62L(L-selection)	Mouse IgG1, κ	DREG-56	304851
TotalSeq™-C0085 anti-human CD25 Antibody	CD25	Mouse IgG1, κ	BC96	302649
TotalSeq™-C0355 anti-human CD137 (4-1BB) Antibody	CD137	Mouse IgG1, κ	4B4-1	309839
TotalSeq™-C0152 anti-human CD223 (LAG-3) Antibody	LAG-3	Mouse IgG1, κ	11C3C65	369335
TotalSeq™-C0176 anti-human CD39 Antibody	CD39	Mouse IgG1, κ	A1	369335
TotalSeq™-C0088 anti-human CD279 (PD-1) Antibody	PD-1	Mouse IgG1, κ	EH12.2H7	329963
TotalSeq™-C0169 anti-human CD366 (Tim-3) Antibody	TIM-3	Mouse IgG1, κ	F38-2E2	345049
TotalSeq™-C0090 Mouse IgG1, κ isotype Ctrl Antibody	Mouse IgG1, κ isotype	Mouse IgG1, κ	MOPC-21	400187
TotalSeq™-C0091 Mouse IgG2a, κ isotype Ctrl Antibody	Mouse IgG2a, κ isotype	Mouse IgG2a, κ	MOPC-173	400293
TotalSeq™-C0092 Mouse IgG2b, κ isotype Ctrl Antibody	Mouse IgG2b, κ isotype	Mouse IgG2b, κ	MPC-11	400381

*Above antibodies were purchased from BioLegend.

Hashtag Antibody	Reactivity	Barcode Sequence	Manufactory	Catalog #
TotalSeq™-C0255 anti-human Hashtag 5 Antibody	Human	AAGTATCGTTTCGCA	BioLegend	394669
TotalSeq™-C0256 anti-human Hashtag 6 Antibody	Human	GGTTGCCAGATGTCA	BioLegend	394671

Supplementary Table 2

Clusters Annotation

Cluster	Cell subtype Annotation	Top Markers	Reference
C0	$CD4^+$ activated T-cells	$CD25^+$ $CD40LG^+$	(Laidlaw et al., 2016)
C9	$CD4^+$ stem memory T-cells (Tscm)	$KLF2^+$ $TCF7^+$	(Gattinoni et al., 2017; Gautam et al., 2019)
C6	Activated effector T-cells ($IFNG^+$ Teff)	$TNFRSF9^+$ IFN-γ^+	(Hamann et al., 1997; Sallusto et al., 1999)
C2	$CD8^+$ resident memory T-cell (Trm)	$CD39^+$ $CD103^+$	(Duhen et al., 2018)
C5	$CD8^+$ T Cycling/G2M	$TOP2A^+$ $MKI67^+$	(Kowalczyk et al., 2015)
C1	CD8+ Early effector memory T-cells (Tem)	GZMK+ CD27+	(Hamann et al., 1997; Mahnke et al., 2013; Sallusto et al., 1999)
C3	CD8+ Late Tem	GZMA+ CD27-	(Hamann et al., 1997; Mahnke et al., 2013; Sallusto et al., 1999)
C7	CD8+ effector T-cells (Teff)	GNLY+ GZMB+	(Kaech and Cui, 2012)
C10	$CD8^+$ central memory T-cells (Tcm)	$KLF2^+$ $SELL^+$	(Hamann et al., 1997; Mahnke et al., 2013; Sallusto et al., 1999)
C14	$CD8^+$ terminally differentiated effector memory (Temra)	$GZMH^+$ $KLRG1^+$	(Henson and Akbar, 2009; Kaech and Cui, 2012)
C11	γδ T-cells	$TRDV2^+$ $TRGV9^+$	(Ribot et al., 2021)
C18	natural killer (NK)-like T-cells	$FCER1G^+$ $TRDC^+$	
C20	LYZ^+ cells	LYZ^+	

Supplementary Data 1

Conserved gene list for each cluster used for cluster annotation. The 'FindAllMarkers()' function was used to compare each cluster against all other clusters to identify potential marker genes. A two-sided non-parametric Wilcoxon rank sum test was performed in this analysis.
