# Supplementary material for: CAR T-cells targeting FGFR4 and CD276 simultaneously show potent antitumor effect against childhood rhabdomyosarcoma
Source: Nat Commun. 2024 Jul 23;15:6222. doi: 10.1038/s41467-024-50251-x (PMC11266617; doi:10.1038/s41467-024-50251-x)
Supplement: Supplementary file 3 — Description of Additional Supplementary Files [file 41467_2024_50251_MOESM3_ESM.pdf]

Description of Additional Supplementary Files

Supplementary Data 1: **Conserved gene list for each cluster used for cluster annotation.** The ‘FindAllMarkers()’ function was used to compare each cluster against all other clusters to identify potential marker genes. A two-sided nonparametric Wilcoxon rank sum test was performed in this analysis.
