# Supplementary material for: CAR T-cells targeting FGFR4 and CD276 simultaneously show potent antitumor effect against childhood rhabdomyosarcoma
Source: Nat Commun. 2024 Jul 23;15:6222. doi: 10.1038/s41467-024-50251-x (PMC11266617; doi:10.1038/s41467-024-50251-x)
Supplement: Supplementary file 5 — Reporting Summary [file 41467_2024_50251_MOESM5_ESM.pdf]

nature portfolio

Corresponding author(s): Javed Khan

Last updated by author(s): May 24, 2024

Reporting Summary

Nature Portfolio wishes to improve the reproducibility of the work that we publish. This form provides structure for consistency and transparency in reporting. For further information on Nature Portfolio policies, see our Editorial Policies and the Editorial Policy Checklist.

Statistics

For all statistical analyses, confirm that the following items are present in the figure legend, table legend, main text, or Methods section.

n/a	Confirmed	
☐	☒	The exact sample size (*n*) for each experimental group/condition, given as a discrete number and unit of measurement
☐	☒	A statement on whether measurements were taken from distinct samples or whether the same sample was measured repeatedly
☐	☒	The statistical test(s) used AND whether they are one- or two-sided<br>*Only common tests should be described solely by name; describe more complex techniques in the Methods section.*
☒	☐	A description of all covariates tested
☐	☒	A description of any assumptions or corrections, such as tests of normality and adjustment for multiple comparisons
☐	☒	A full description of the statistical parameters including central tendency (e.g. means) or other basic estimates (e.g. regression coefficient) AND variation (e.g. standard deviation) or associated estimates of uncertainty (e.g. confidence intervals)
☐	☒	For null hypothesis testing, the test statistic (e.g. *F*, *t*, *r*) with confidence intervals, effect sizes, degrees of freedom and *P* value noted<br>*Give P values as exact values whenever suitable.*
☒	☐	For Bayesian analysis, information on the choice of priors and Markov chain Monte Carlo settings
☒	☐	For hierarchical and complex designs, identification of the appropriate level for tests and full reporting of outcomes
☒	☐	Estimates of effect sizes (e.g. Cohen's *d*, Pearson's *r*), indicating how they were calculated

Our web collection on statistics for biologists contains articles on many of the points above.

Software and code

Policy information about availability of computer code

Data collection	Illumina NextSeq500 sequencer was used for CTIE-seq.<br>BioRad ChemiDoc MP 2.3.0.07 was used to obtain images of Western Blots.<br>xCELLigence® real-time cell analysis (RTCA) was used to test the killing ability of CAR T-cells against tumor cells.<br>BD LSRFortessa™ or FACSymphony A5 (BD Bioscience) Cell Analyzers using BD FACS DIVA software v8.0.1 were used for flow cytometry data collection.<br>BD FACSAria Fusion or a BD FACSAria UV Cell Sorters were used for T-cell sorting.<br>IVIS Lumina III In Vivo Imaging system.
Data analysis	CITE-seq: Reads were aligned with human reference genome version GRCh38 and CAR binder scFv region sequences and quantified with Cell Ranger 7.0.1 (10× Genomics) using the standard workflow. The Cell Ranger raw output was imported into R (v4.2.1) using the Seurat package (v 4.3.0) to be analyzed. A custom code for CITE-seq analyses is available on github at https://github.com/CCRGeneticsBranch/Khanlab_CITEseq_Tumor-Infiltrating-CART.<br>GraphPad Prism 8.0 was used for statistical analysis.<br>Cytotoxicity assay data were analyzed using RTCA Software 2.0 (Acea Biosciences).<br>BioRad Image Lab 6.1 software was used to quantitate Western blot bands.<br>FlowJo v10.7.2 software was used for FACS data analysis.<br>Bioluminescent signal flux was quantified using Living Image software (PerkinElmer, Waltham, MA, USA) version 4.5.4.

For manuscripts utilizing custom algorithms or software that are central to the research but not yet described in published literature, software must be made available to editors and reviewers. We strongly encourage code deposition in a community repository (e.g. GitHub). See the Nature Portfolio guidelines for submitting code & software for further information.

Data

Policy information about availability of data

All manuscripts must include a data availability statement. This statement should provide the following information, where applicable:

- Accession codes, unique identifiers, or web links for publicly available datasets
- A description of any restrictions on data availability
- For clinical datasets or third party data, please ensure that the statement adheres to our policy

Raw and processed data from the scRNA, and surface protein sequencing experiments are deposited in the NCBI's Gene Expression Omnibus (GEO, RRID: SCR_005012) database (under accession code GEO GSE228127). [https://www.ncbi.nlm.nih.gov/geo/query/acc.cgi?acc=GSE228127]. Publicly available ChIP-seq data used in this study are available in the GEO database under accession codes GSE83728 [https://www.ncbi.nlm.nih.gov/geo/query/acc.cgi?acc=GSE83728] and GSE116344 [https://www.ncbi.nlm.nih.gov/geo/query/acc.cgi?acc=GSE116344]. The remaining data are available within the Article, Supplementary Information or Source Data files. Source data are provided with this paper.

No new software was developed to perform analysis on the samples.

Our analysis pipeline utilizes well established tools in literature. To be completely transparent, we have published our analysis pipeline on Github and they are referenced in the paper: https://github.com/CCRGeneticsBranch/Khanlab_CITEseq_Tumor-Infiltrating-CART.

Research involving human participants, their data, or biological material

Policy information about studies with human participants or human data. See also policy information about sex, gender (identity/presentation), and sexual orientation and race, ethnicity and racism.

Reporting on sex and gender	No human participants were involved in this study.
Reporting on race, ethnicity, or other socially relevant groupings	No human participants were involved in this study.
Population characteristics	No human participants were involved in this study.
Recruitment	No human participants were involved in this study.
Ethics oversight	No human participants were involved in this study.

Note that full information on the approval of the study protocol must also be provided in the manuscript.

Field-specific reporting

Please select the one below that is the best fit for your research. If you are not sure, read the appropriate sections before making your selection.

☒ Life sciences ☐ Behavioural & social sciences ☐ Ecological, evolutionary & environmental sciences

For a reference copy of the document with all sections, see nature.com/documents/nr-reporting-summary-flat.pdf

Life sciences study design

All studies must disclose on these points even when the disclosure is negative.

Sample size	Three to seven mice were used in every treatment or control arm in all mouse experiments to have statistical power. For in vitro studies, sample size of at least 3 was used in each experiment. Sample size and number of independent experiments are stated in the figure legends.
Data exclusions	No data points was excluded to draw conclusions.
Replication	Three replicates were successfully performed to ensure data reproducibility for all in-vitro experiments. For in vivo mouse experiments, all the experiments were repeated using at least two different T cell donors with at 3-7 mice in each group, each mice represents one repeat. The replication of experiments showed similar results or trend.
Randomization	All tumor bearing mice were placed into one cage and allowed to mix. Then mice were randomly selected one at a time and placed into cage for control versus treatment in alternating order. In vitro studies were performed using cell lines and we did not randomize. Covariates were controlled by performing the experiment at the same time using the same growth media, flasks, reagents, kits, data collecting machine.
Blinding	Investigators were not blinded for treatment versus control. Blinding is not feasible in the experiments described here due the treatment and follow up schedule.

Reporting for specific materials, systems and methods

We require information from authors about some types of materials, experimental systems and methods used in many studies. Here, indicate whether each material, system or method listed is relevant to your study. If you are not sure if a list item applies to your research, read the appropriate section before selecting a response.

Materials & experimental systems

n/a	Involved in the study
☐	☒ Antibodies
☐	☒ Eukaryotic cell lines
☐	☒ Palaeontology and archaeology
☐	☒ Animals and other organisms
☒	☐ Clinical data
☒	☐ Dual use research of concern
☒	☐ Plants

Methods

n/a	Involved in the study
☒	☐ ChIP-seq
☐	☒ Flow cytometry
☒	☐ MRI-based neuroimaging

Antibodies

Antibodies used

Mouse anti-human FGFR4 antibody, clone 3A11 (in-housed, 1 μg/ml)
Recombinant humanized anti-human CD276 antibody, clone MGA271 (Creative Biolabs, Cat# TAB-117CL, 1 μg/ml)
PE-conjugated Goat anti-mouse immunoglobulin G (IgG) antibody (Biolegend, Cat# 405307, 1:300)
R-Phycoerythrin AffiniPure F(ab')2 Fragment Goat Anti-Human IgG, Fcγ Fragment specific (Jackson ImmunoResearch Laboratories, Cat# 109-116-170, 1:300)
Ultra-LEAFTM Purified Mouse IgG1, k isotype control antibody, Clone MG1-45V (Biolegend, Cat# 401414, 1 μg/ml)
Ultra-LEAFTM Purified Human IgG1 Isotype Control Recombinant Antibody, Clone QA16A12 (Biolegend, Cat# 403502, 1 μg/ml)
FITC anti-human CD45 antibody, Clone HI30 (BioLegend, Cat# 304006, 1:100)
PE anti-human EGFR antibody , clone AY13 (BioLegend, Cat# 352904, 1:50)
PerCP/Cyanine5.5 anti-human CD62L Antibody, Clone DREG-56 (BioLegend, Cat# 304824, 1:100)
PE/Cyanine7 anti-human CD366 (Tim-3) Antibody, Clone F38-3E2 (BioLegend, Cat# 345014, 1:100)
PE-Dazzle594 anti-human CD4 antibody, Clone A161A1 (BioLegend, Cat# 357412, 1:100)
APC anti-human CD8a antibody, clone RPA-T8 (BioLegend, Cat# 301049, 1:100)
Alexa Fluor® 700 anti-human CD3 Antibody, Clone OKT3 (BioLegend, Cat# 317340, 1:100)
APC/Cyanine7 anti-human CD39 Antibody, Clone A1 (BioLegend, Cat# 328226, 1:100)
Brilliant Violet 421™ anti-human CD223 (LAG-3) Antibody, Clone 11C3C65 (BioLegend, Cat# 369314, 1:100)
Brilliant Violet 605™ anti-human CD45RA Antibody, Clone HI100 (BioLegend, Cat# 304134, 1:100)
Brilliant Violet 711™ anti-human CD274 (B7-H1, PD-L1) Antibody, Clone EH12.2H7 (BioLegend, Cat# 329722, 1:100)
BD Horizon™ BUV737 Mouse Anti-Human CD8, Clone SK1 (BD, Cat# 612755, 1:400)
Fixable viability dye Ghost Dye™ Violet 510 (TONBO, Cat#13-0870-T100, 1:100)
Recombinant human FGFR4-Fc Chimera Protein (Abcam, Cat# ab83999, 1μg/ml)
Biotinylated Human B7-H3 (4Ig)-Fc Chimeric Protein (Acro Biosystem, Cat# B73-H82F5, 1μg/ml)
R-Phycoerythrin AffiniPure F(ab')2 Fragment Goat Anti-Human IgG, Fcγ Fragment specific (Jackson ImmunoResearch Laboratories, Cat# 109-116-170, 1:300)
AF647 conjugated Fab specific for human IgG-Fc (Jackson ImmunoResearch Laboratories, Cat# 109-607-008, 1:300)
TotalSeq™-C0034 anti-human CD3 Antibody, Clone UCHT1 (BioLegend, Cat# 300479, 0.5μg per million cells)
TotalSeq™-C0072 anti-human CD4 Antibody, Clone RPA-T4 (BioLegend, Cat# 300567, 0.5μg per million cells)
TotalSeq™-C0046 anti-human CD8 Antibody, Clone RPA-T8 (BioLegend, Cat# 344753, 0.5μg per million cells)
TotalSeq™-C0063 anti-human CD45RA Antibody, Clone HI100 (BioLegend, Cat# 304163, 0.5μg per million cells)
TotalSeq™-C0087 anti-human CD45RO Antibody, Clone UCHL1 (BioLegend, Cat# 304259, 0.5μg per million cells)
TotalSeq™-C0154 anti-human CD27 Antibody, Clone O323 (BioLegend, Cat# 302853, 0.5μg per million cells)
TotalSeq™-C0156 anti-human CD95 (Fas) Antibody, Clone DX2 (BioLegend, Cat# 305651, 0.5μg per million cells)
TotalSeq™-C0147 anti-human CD62L Antibody, Clone DREG-56 (BioLegend, Cat# 304851, 0.5μg per million cells)
TotalSeq™-C0085 anti-human CD25 Antibody, Clone BC96 (BioLegend, Cat# 302649, 0.5μg per million cells)
TotalSeq™-C0355 anti-human CD137 (4-1BB) Antibody, Clone 4B4-1 (BioLegend, Cat# 309839, 0.5μg per million cells)
TotalSeq™-C0152 anti-human CD223 (LAG-3) Antibody, Clone 11C3C65 (BioLegend, Cat# 369339, 0.5μg per million cells)
TotalSeq™-C0176 anti-human CD39 Antibody, Clone A1 (BioLegend, Cat# 328237, 0.5μg per million cells)
TotalSeq™-C0088 anti-human CD279 (PD-1) Antibody, Clone EH12.2H7 (BioLegend, Cat# 329963, 0.5μg per million cells)
TotalSeq™-C0169 anti-human CD366 (Tim-3) Antibody, Clone F38-2E2 (BioLegend, Cat# 345049, 0.5μg per million cells)
TotalSeq™-C0090 Mouse IgG1, κ isotype Ctrl Antibody, Clone MOPC-21 (BioLegend, Cat# 400187, 0.5μg per million cells)
TotalSeq™-C0091 Mouse IgG2a, κ isotype Ctrl Antibody, Clone MOPC-173 (BioLegend, Cat# 400293, 0.5μg per million cells)
TotalSeq™-C0092 Mouse IgG2b, κ isotype Ctrl Antibody, Clone MPC-11 (BioLegend, Cat# 400381, 0.5μg per million cells)
TotalSeq™-C0255 anti-human Hashtag 5 Antibody, Clone LNH-94,2M2 (BioLegend, Cat# 394669, 0.5μg per million cells)
TotalSeq™-C0256 anti-human Hashtag 6 Antibody, Clone LNH-94,2M2 (BioLegend, Cat# 394671, 0.5μg per million cells)
Recombinant Anti-CD3 zeta (phospho Y142) antibody, Clone EP265(2)Y, (Abcam, Cat# ab68235, 1:1000)
anti-CD3ζ, Clone 6C10.2 (Santa Cruz Biotechnology, Cat# SC-1239, 1:2000)
Rabbit anti-Phospho-Zap-70 (Tyr319)/Syk (Tyr352), Clone 65E4 (Cell Signaling Technology, Cat# 2717, 1:1000)
Mouse anti-Zap-70, Clone L1E5 (Cell Signaling Technology, Cat# 2709, 1:1000);
Rabbit anti-Phospho-PLCγ1 (Tyr783), Clone D6M9S (Cell Signaling Technology, Cat #14008, 1:1000),
Rabbit anti-PLCγ1, Clone D9H10 (Cell Signaling Technology, Cat#5690, 1:1000)

	Rabbit anti-Phospho-p44/42 MAPK (Erk1/2) (Thr202/Tyr204), Clone D13.14.4E (Cell Signaling Technology, Cat# 4370, 1:2000)<br>p44/42 MAPK (Erk1/2) Antibody (Cell Signaling Technology, Cat# 9102, 1:2000)<br>Rabbit anti-phospho-Akt (Ser473), Clone D9E (Cell Signaling Technology, Cat# 4060, 1:2000)<br>Rabbit anti-Akt (pan), Clone C67E7 (Cell Signaling Technology, Cat# 4691, 1:2000)<br>Rabbit anti-Phospho-NF-κB p65 (Ser536), Clone 93H1 (Cell Signaling Technology, Cat# 3033, 1:1000)<br>Rabbit anti-NF-κB p65, Clone D14E12 (Cell Signaling Technology, Cat# 8242, 1:1000)<br>anti-mouse-HRP (Cell Signaling Technology, Cat# 7076, 1:5000)<br>anti-rabbit-HRP (Cell Signaling Technology, Cat# 7074, 1:5000)
Validation	All antibodies used were validated by the suppliers for their particular application. Validation statements and relevant references for the used antibodies can be found on the manufacture's website.

Eukaryotic cell lines

Policy information about cell lines and Sex and Gender in Research

Cell line source(s)	Human RMS cell lines RH30, RH5, and RH4 were all provided by Dr. Peter Houghton, Greehey Children's Cancer Research Institute, San Antonio, Texas, USA. JR was a gift from Dr. Corinne M. Linardic at Duke University School of Medicine, Durham, NC, USA.RMS559 was obtained from Dr. Jonathan Fletcher at Brigham and Women's Hospital, Boston, USA. RD, CTR, and BIRCH were provided by Dr. Lee Helman, Children's Hospital Los Angeles, CA, USA. SCMC was provided by Dr. Janet Shipley, Institute of Cancer Research, London, England. GFP/Luciferase over-expressed RH30, RMS559, JR, and FGFR4-KO RH30,D276-KO RH30 cell lines were generated in our lab.<br>The patient-derived xenografts used in this study, including SJRHB013759_X1 (FP-RMS), RMS-ZH003 (FP-RMS), SJRHB013758 (FNRMS), RMS-ZH010 (FN-RMS), SJRHB000026_X2 (FN-RMS), SJRHB013_X (FN-RMS), and SJRHB015720_X1 (MYOD1 Mutant RMS), were obtained from St. Jude Children's research hospital (Memphis, Tennessee, USA).
Authentication	STR profiling was used for cell line authentication.
Mycoplasma contamination	All cell lines tested negative for Mycoplasma contamination.
Commonly misidentified lines<br>(See ICLAC register)	No commonly misidentified cell lines were used in this study.

Palaeontology and Archaeology

Specimen provenance	*Provide provenance information for specimens and describe permits that were obtained for the work (including the name of the issuing authority, the date of issue, and any identifying information). Permits should encompass collection and, where applicable, export.*
Specimen deposition	*Indicate where the specimens have been deposited to permit free access by other researchers.*
Dating methods	*If new dates are provided, describe how they were obtained (e.g. collection, storage, sample pretreatment and measurement), where they were obtained (i.e. lab name), the calibration program and the protocol for quality assurance OR state that no new dates are provided.*

☐ Tick this box to confirm that the raw and calibrated dates are available in the paper or in Supplementary Information.

Ethics oversight	*Identify the organization(s) that approved or provided guidance on the study protocol, OR state that no ethical approval or guidance was required and explain why not.*

Note that full information on the approval of the study protocol must also be provided in the manuscript.

Animals and other research organisms

Policy information about studies involving animals; ARRIVE guidelines recommended for reporting animal research, and Sex and Gender in Research

Laboratory animals	5-7 weeks of female NOD/SCID/IL-2Rgcnull (NSG) mice were used in this study. Only female mice were used in the study.
Wild animals	This study did not involve wild animals
Reporting on sex	Female mice were used in this study which allows for housing of 5 mice per cage. Sex consideration was based on practical feasibility issues. This included aggressiveness of male mice with propensity to injure one another which is especially concerning in severely immunocompromised NSG mice. Mice are housed in IVM Microisolator conditions designated for immune compromised mice. Light cycle is 6am-6pm.
Field-collected samples	This study did not involve field collected samples
Ethics oversight	This mouse study was reviewed and was approved by the Institutional Animal Care and Use Committee(IACUC) at the NIH, protocol GB-011.

Note that full information on the approval of the study protocol must also be provided in the manuscript.

Plants

Seed stocks	N/A
Novel plant genotypes	N/A
Authentication	N/A

Flow Cytometry

Plots

Confirm that:

☒ The axis labels state the marker and fluorochrome used (e.g. CD4-FITC).

☒ The axis scales are clearly visible. Include numbers along axes only for bottom left plot of group (a 'group' is an analysis of identical markers).

☒ All plots are contour plots with outliers or pseudocolor plots.

☒ A numerical value for number of cells or percentage (with statistics) is provided.

Methodology

Sample preparation	Samples were stained in DPBS supplemented with 2% heat inactivated FBS and 2mM EDTA with different antibody dilutions.
Instrument	BD LSRFortessa™ or FACSymphony A5 (BD Bioscience) Cell AnalyzersCell Analyzer
Software	FACSDiva software was used for data acquisition and FlowJo software was used for data analysis.
Cell population abundance	A minimum of 100k single cells were analyzed per sample.
Gating strategy	Cell debris and cell doublets were removed from analysis using FSC and SSC gates, and then gate out from viability dye stained population. CAR+(FGFR4-Fc or CD276-Biotin stained positive) lymphocytes were gated on live CD45+ cells. T cell exhaustion and differentiation were analyzed in the CD3+CAR+ T cell population.

☒ Tick this box to confirm that a figure exemplifying the gating strategy is provided in the Supplementary Information.
